# Insights into early generation synthetic amphidiploid *Brassica juncea*: a strategy to harness maximum parental genomic diversity for improving Indian mustard

**DOI:** 10.3389/fpls.2025.1493618

**Published:** 2025-02-13

**Authors:** Pooja Garg, Shikha Tripathi, Anamika Kashyap, A. Anil Kumar, Sujata Kumari, Mandeep Singh, Ranjeet Kushwaha, Shiv Shankar Sharma, Jyoti Sharma, Rashmi Yadav, N. C. Gupta, Naveen Singh, Ramcharan Bhattacharya, Vinod Chhokar, Mahesh Rao

**Affiliations:** ^1^ Indian Council of Agricultural Research (ICAR)- National Institute for Plant Biotechnology (NIPB), New Delhi, India; ^2^ Department of Biotechnology, Guru Jambheshwar University of Science and Technology (GJUS&T), Hisar, Haryana, India; ^3^ Department of Botany, Institute of Science, Banaras Hindu University (BHU), Varanasi, Uttar Pradesh, India; ^4^ Veer Chandra Singh Garhwali Uttarakhand University of Horticulture & Forestry, Bharsar, Uttarakhand, India; ^5^ Crop Improvement Section, ICAR-Indian Institute of Oilseeds Research, Hyderabad, Telangana, India; ^6^ Division of Genetics, ICAR-Indian Agricultural Research Institute (IARI), New Delhi, India; ^7^ Applied Genomics Section, Bhabha Atomic Research Centre (BARC), Mumbai, India; ^8^ Division of Germplasm Evaluation, ICAR-National Bureau of Plant Genetic Resources (NBPGR), New Delhi, India

**Keywords:** *Brassica juncea*, inter-specific hybridization, resynthesized lines, genetic diversity, SSR markers, pollen fertility, allelic richness

## Abstract

In India, amphidiploid *Brassica juncea* (AABB, 2n=36) is a significant oilseed crop, but its small gene pool limits its ability to develop traits of higher breeding and economic value. Through interspecific hybridization from various lines of the progenitor species, resynthesized *B. juncea* (RBJ) can provide breeders with additional resources for creating genetically diverse stress-tolerant and high-yielding cultivars. Three *B. rapa* accessions and eight *B. nigra* accessions were crossed in this study to develop 33 synthetic *B. juncea* lines. A total of 28 crosses were attempted, including the three-way crosses, but only the cross combinations with *B. rapa* cytoplasm led to successful embryonic development. Molecular diversity analysis of these lines in S_2_ generation revealed significant genetic diversity with higher levels of heterozygosity and allelic richness, along with significant variations for the yield-related traits. These results suggest that the synthesized lines could effectively enrich the genetic base of *B. juncea* and generate variability for agronomically important traits in a shorter time duration. The characterized variability in the synthetic lines needs to be utilized in hybridization, with already evolved genotypes, in early generations before it is lost due to chromosomal rearrangements, recombination and natural selection.

## 1 Introduction

Amphidiploid *Brassica juncea* holds a pivotal position as a vital oilseed crop in India and adjacent Asian countries, making a substantial contribution to the agricultural landscape of these countries. Specifically in India, 23.5% of the cultivated area is dedicated to oilseed crops and is responsible for approximately 24.2% of the total oilseed production within the country (Jat et al., 2019). The allotetraploid species, *B. juncea* has evolved from the hybridization of two different diploid progenitor species- *B. rapa* (AA, 2n = 20) and *B. nigra* (BB, 2n = 16), encompassing genetic diversity from both the progenitors and, potentially creating a genetic base for the developing cultivars (Jat et al., 2019). However, complex polyploidy, selective domestication, and modern breeding techniques restricted the genetic variability in Indian mustard *(B. juncea)* (Li et al., 2013; Zhang et al., 2022). It is also anticipated that during the process of evolution, the genetic diversity of both the parental species is not fully utilized, resulting in a narrow genetic base in the natural gene pool, ultimately limiting the potential for breeders to develop new promising cultivars with desired traits (Gepts and Papa, 2003; Hu et al., 2021; Singh et al., 2021).

One of the valuable approaches for developing a new gene pool is resynthesizing allotetraploid species that gives access to a new genetic diversity that may or may not exist in the original parental species, conferring desirable traits such as yield contributing traits, quality parameters, and tolerance to different biotic and abiotic stresses. This will allow the breeders to utilize the diverse parental diploid progenitor species to develop a diverse set of genetic stocks of *B. juncea* which will enrich its primary gene pool. This technique typically involves crossing the ancestral parental species and then inducing polyploidization in the resulting hybrid (Mohd Saad et al., 2021; Quezada-Martinez et al., 2021; Hu et al., 2021), which can be done through various means, such as chemical treatment or by crossing the hybrid with a closely related tetraploid species followed by the screening for desirable traits (Li et al., 2018; Petereit et al., 2022). This technique of upsurging genetic diversity provides the breeders with more opportunities to create cultivars for sustainable production of *B. juncea* and other allotetraploid species, benefiting the agriculture industry and food security (Parmar et al., 2017; Mohd Saad et al., 2021).

Previously, synthetic lines have been successfully resynthesized in various crop species to improve genetic diversity and enhance desirable traits (Eduardo et al., 2020). For instance, in wheat (*Triticum aestivum*), the resynthesis of hexaploid wheat was accomplished by crossing tetraploid durum wheat (*T. turgidum*) with diploid *Aegilops tauschii*, resulting in novel genotypes with improved yield potential and resistance to biotic and abiotic stresses (Ogbonnaya et al., 2013). All three amphidiploid species were resynthesized and utilized to generate new genetic variability and recover desirable traits in *Brassica* crops. The resynthesized *B. napus* and *B. carinata* lines have been used as a source of genetic diversity in breeding programs to improve the yield and quality of *Brassica* crops and to enhance their resistance to biotic and abiotic stresses (Ozminkowski and Jourdan, 1994; Zhang et al., 2004; Rahman et al., 2015).

The previous studies by Bansal et al. (2009); Katiyar et al. (1998); Yadav et al. (2009), and Sheng et al. (2012) have also utilized diploid progenitor species of *B. juncea* to generate variation in morpho-physiological traits. We want to highlight that most of the earlier efforts on re-synthesis of *B. juncea* have involved *B. rapa* variety yellow sarson due to high recovery/efficiency (Hinata and Konno, 1979; Bhat and Sarla, 2004). Owing to the poor efficiency and self-incompatible nature of *B. rapa* var. toria, it was rarely successful in developing synthetic *B. juncea* (Srinivasachar, 1964; Prakash, 1973). In this study, we have followed a new approach along with the earlier method, wherein *B. rapa* var. yellow sarson was crossed with *B. rapa* var. toria and the F_1_s derived from these crosses (including reciprocal) were mated with B genome donor (*B. nigra*). This will allow the unique genetic variability available in *B. rapa* var. toria to tap into resynthesizing *B. juncea*. This study also addresses the challenges associated with breeding synthetic *B. juncea* lines, i.e., tissue culture being the most time-consuming and tedious aspect of resynthesis. Here, crosses are attempted using *B. rapa* var. yellow sarson NRCPB rapa 8 (IC0623820), a novel *B. rapa* germplasm that bypasses the need to rescue the embryos and hence, tissue culture interventions are not needed (Rao et al., 2024). Furthermore, we noticed that in most of the earlier reports, one or few accessions of *B. nigra* were involved for this purpose (Prakash, 1973; Bhat and Sarla, 2004; Sheng et al., 2012), and therefore, we utilized the varied *B. nigra* accessions to generate the synthetic lines. Thus, a highly diverse set of materials and a novel approach were deployed for developing synthetic *B. juncea* lines. We propose that the observed diversity in the early generation provides valuable insights for mustard breeding programs, especially when aiming to exploit the maximum genetic potential inherited from the parental species. Genetic variability lying dormant in the synthetic lines needs to be characterized and utilized for hybridization with the already evolved *B. juncea* genotypes before it is lost due to the bottleneck effect of populations, chromosomal rearrangements, recombination, natural selection, etc. Therefore, we characterized the developed synthetic lines in the S_2_ generation to capture the maximum diversity inherited from the parental species. The main aim of this study was to (i) create, report and harness novel genetic variability for yield contributing traits at early generation, which was lost during domestication/evolution of *B. juncea*, and (ii) explore a novel approach for involving *B. rapa* var. toria (*B. rapa* var. yellow sarson/*B. rapa* var. toria/*B. nigra*) in developing synthetic *B. juncea.*

## 2 Materials and methods

### 2.1 Plant materials

**Figure 1:**
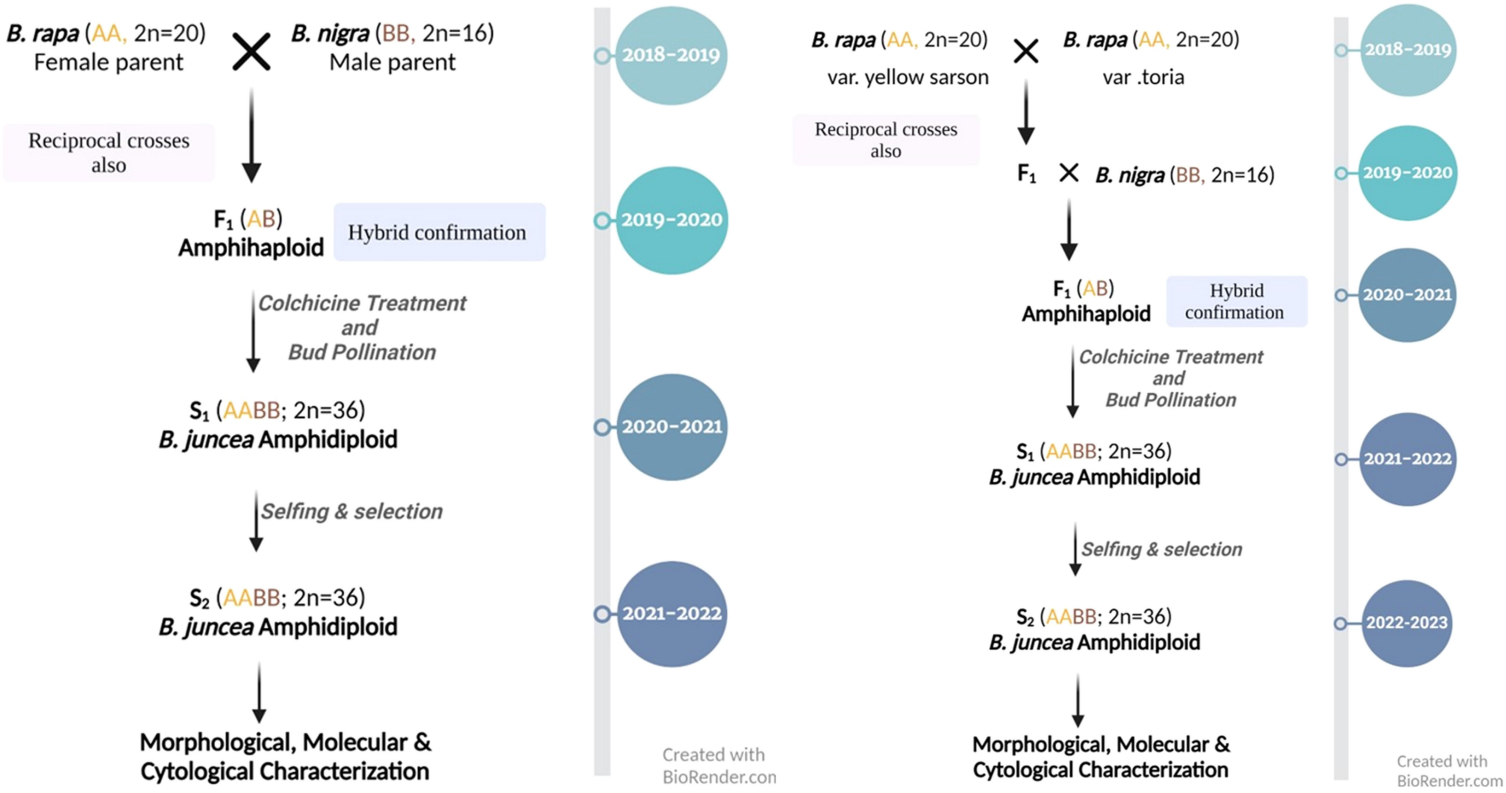
Year-wise scheme opted for synthesis of *B*. *juncea* by hybridization of *B*. *rapa* with *B*. *nigra* (left) and involving *B*. *rapa* var. yellow sarson, *B*. *rapa* var. toria and *B*. *nigra* in three-way crosses (right).

Three accessions of *B. rapa* (var. yellow sarson and toria) and eight *B. nigra* were used for inter-specific crosses to develop resynthesized *B. juncea* (RBJ) (scheme in **Figure 1**), as listed in **Table 1**. A total of 28 cross combinations (**Supplementary Table S1**) were attempted using different accessions of *B. rapa* and *B. nigra* as both male and female parents. To infuse larger genetic variability in resynthesized lines, a three-way cross approach was used in which *B. rapa* var. yellow sarson and *B. rapa* var. toria were hybridized to generate F_1_ seed for further crossing with *B. nigra* populations. *B. juncea* cultivars, namely, Pusa Jaikisan (PJK), Pusa Vijay (P. Vijay), Pusa Mustard 28 (PM 28) and Varuna were used as controls to compare with the synthetic lines developed.

**Table 1:** List of *B. rapa* and *B. nigra* accessions used in the study for interspecific hybridization.

Germplasm	Accessions used
*Brassica rapa*	Rapa 12 (var. toria), NRCPB rapa 8 (IC0623820) (var. yellow sarson), Pusa gold (var. yellow sarson)
*Brassica nigra*	Nigra tall, Nigra 2 (EC426390), IC 338498, IC 338724, IC 341132, IC 393266, IC 399882, IC 328460

### 2.2 Emasculation and pollination

The mature, unopened buds were chosen for emasculation to avoid any self or foreign pollen contamination. All the anthers, sepals, and petals were removed collectively from the selected flower buds using forceps, adjoining younger buds were snipped off, and the inflorescence was bagged. The male parent’s inflorescence was bagged to avoid any pollen contamination (Seyis et al., 2005). The following day, collected pollens from the male parent were used to pollinate the female parent’s emasculated buds.

### 2.3 Chromosome doubling by colchicine treatment

To develop amphidiploids from amphihaploid inter-specific hybrids, non-absorbent cotton balls soaked in 0.2% colchicine were applied to the hybrid plants’ axillary buds and apical meristem on alternate days for 5-7 days (Rajcan et al., 2011; Manzoor et al., 2019).

### 2.4 Pollen viability test

The viability of pollens in the parental and interspecific hybrid plants was examined under a light microscope (Carl Zeiss Axiolab 5, Germany) using the protocol by Katche et al. (2021). The mature flower buds were collected in the ice container, and with the aid of forceps and a needle, the anther was removed and crushed in 1% acetocarmine. Three buds from each plant were inspected to determine the pollen viability, which was analyzed and expressed in terms of percentage. Round, plump, and stained pollens were regarded as viable, whereas shriveled or unstained pollens were sterile.

### 2.5 Cytogenetic analysis

The procedure of Snowdon et al. (1997) was followed to count the total number of diploid (2n) chromosomes in mitotic cells from root tissue by applying DAPI (4,6-diamidino-2-phenylindole) as fluorescent dye under the microscope. The root tips were harvested early in the morning in 0.002M 8-hydroxyquinoline solution and then fixed in Carnoy’s solution (3:1 ethanol: acetic acid solution), followed by transfer into 70% ethanol. For slide preparation, enzyme solution was added to root tips on a slide and incubated at 37°C for 45 minutes, followed by the addition of 10µL 45% acetic acid after removing the enzyme solution (Rao et al., 2024). The root tip was solubilized with a needle to release the cells, and then a coverslip was placed on top. The slide was fixed in liquid N_2,_ and the coverslip was removed with the help of a scalpel blade and then allowed to air dry. After staining with 10µL DAPI, the chromosomes were seen under the fluorescent microscope (Carl Zeiss Axiolab 5, Germany).

### 2.6 Evaluation of morphological traits

Phenotypic data was collected from three selected plants in S_2_ generation of each line across the three replication plots for plant height (PH), main shoot length (MSL), silique length (SL), number of siliques on main shoot (SMS), number of primary branches (PB), seeds per silique (SS), oil content (OC), yield per plant (YP), and thousand seed weight (TSW). Oil content was estimated using the Near-Infrared Spectroscopy (NIRS) by Newport NMR analyzer (Model-4000) (Shruti et al., 2023). The morphological diversity was assessed for thirty-three synthetic *B. juncea* lines, four *B. juncea* cultivars, and diploid parental species, i.e., *B. rapa* (3 accessions) and *B. nigra* (8 accessions).

### 2.7 Confirmation of hybridity and molecular diversity analysis

A set of 94 SSR markers (**Supplementary Table S3**) pertaining to AA (*B. rapa*), BB (*B. nigra*) and AABB (*B. juncea*) genome were selected for the hybridity confirmation (**Supplementary Table 3**) in F_1_ generation along with genetic diversity analysis in S_2_ generation (Sudan et al., 2016). These polymorphic markers are available in the Brassicaceae database (BRAD) (http://Brassicadb.cn) (Chen et al., 2022) and are also reported by Lowe et al. (2004); Kim et al. (2009); Dhaka et al. (2017).

The total genomic DNA was isolated following DNA extraction protocol from fresh leaves (Doyle and Doyle, 1990). In a reaction volume of 20 µl, the PCR mixture contained 1 µl of template DNA (25 ng/µl), 1 µl of each forward and reverse primer (100 pmol/µl), 1µl of 10 mM dNTPs, 1.5 µl of 25mM MgCl_2_, 4 µl of 10x PCR buffer, and 0.5µl of 0.5U *Taq* polymerase and 10 µl of nuclease-free water. The PCR cycle was designed with an initial denaturation at 94°C for 5 min, followed by 35 cycles of denaturation at 94°C for 1 min, annealing at 54°C for 1 min and 15 sec, and extension at 72°C for 1 min 30 sec, before a final extension at 72°C for 10 min. The PCR products were processed in 1x TAE buffer and separated on a 2.5% agarose gel along with the 50 bp DNA ladder as a benchmark on both sides of the gel.

### 2.8 Data analysis

The statistical analysis was done using the metan package in R program v4.2.0 (Olivoto and Lúcio, 2020). Due to the presence of multi-collinear factors, the data was subjected to principal component analysis (PCA) based clustering. While conducting a cluster analysis with pair group distance and Euclidean similarity metrics, the factors corresponding to significant PCs were chosen. Using the DARwin software v6.0.021, the neighbor-joining tree was created (Perrier and Jacquemoud-Collet, 2006). The population structure was assessed using the Bayesian clustering model-based software STRUCTURE v2.3.4 (Pritchard et al., 2000; Falush et al., 2003). Five iterations were performed for each cluster, *K* = 2 to 8, with the length of the burn-in period and Markov Chain Monte Carlo (MCMC) replications set to 50,000 each. The most probable *K* value was determined using a web-based software StructureSelector (Li and Liu, 2018), which uses combined measures and estimators to select the best *K*-value (Evanno et al., 2005; Raj et al., 2014; Puechmaille, 2016) and integrates CLUMPAK program for graphical representation (Kopelman et al., 2015).

## 3 Results

### 3.1 *B. rapa* as the female parent yielded successful cross combinations with intermediate parental phenotypes in the progenies

**Figure 2:**
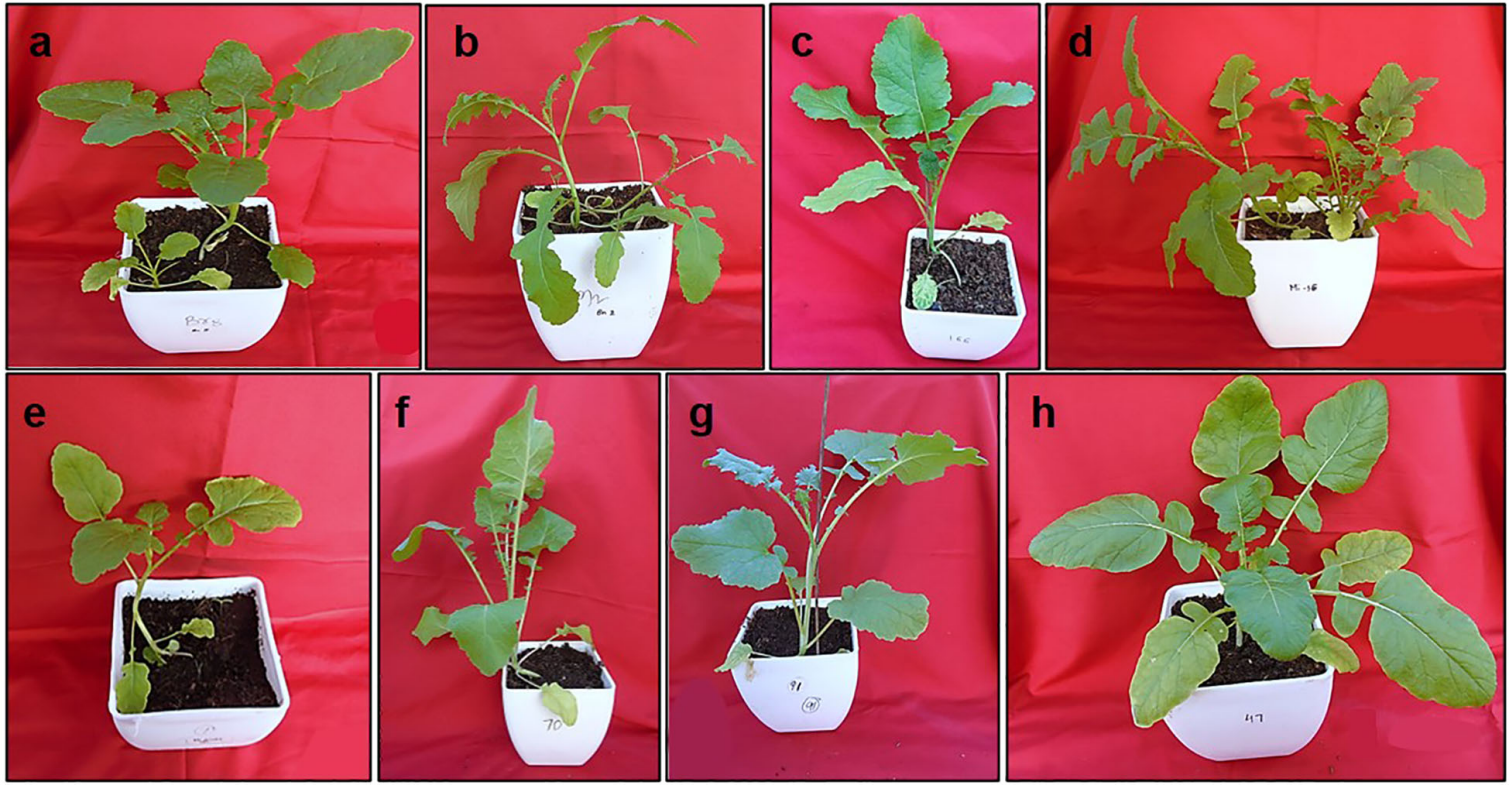
Morphology of plants under controlled conditions **(A)** NRCPB rapa 8 (female parent), **(B)** *B*. *nigra* Dwarf (male parent), **(C)** Pusa Jaikisan (cultivar), **(D–F),** F_1_ and **(G, H)** S_1_ generations of synthesized *B*. *juncea* lines (RBJ 106, RBJ 135).

**Figure 3:**
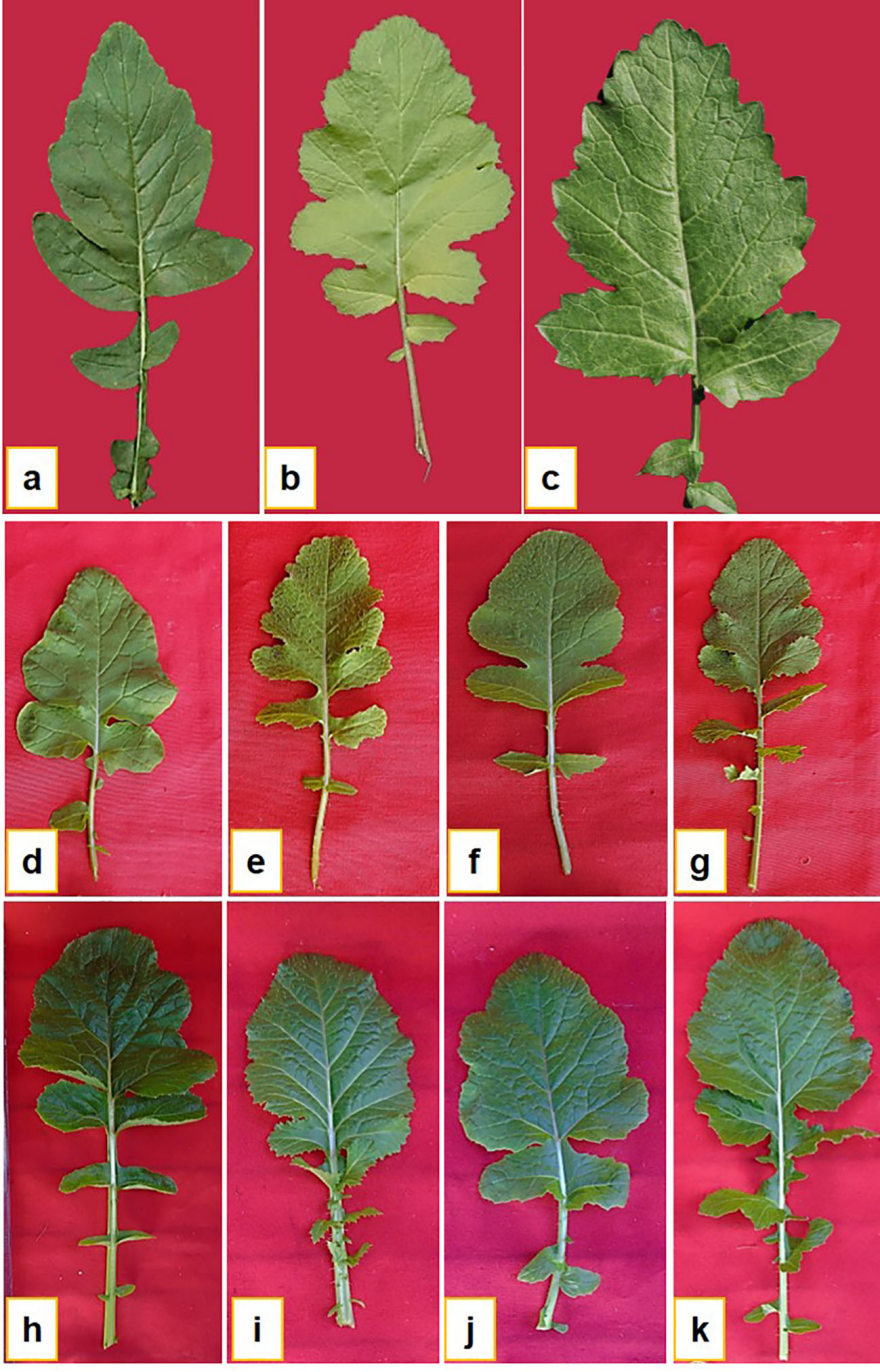
Leaf morphology of **(A)** NRCPB rapa 8 (female parent), **(B)** *B*. *nigra* Dwarf (male parent), **(C)** Pusa Jaikisan (cultivar), **(D–G)** F_1_ and **(H–K)** S_1_ generations of synthesized *B*. *juncea* lines (RBJ104, RBJ 119, RBJ 126, RBJ 147).

Twenty-eight crosses attempted in this study yielded thirty-three lines of RBJ in twelve cross combinations, as given in **Table 2**. The crosses were successful for combinations having *B. rapa* as the female parent, and hence, no seeds were obtained for reciprocal crosses (*B. nigra* ×*B. rapa*). When compared with parents and control (**Figures 2A–C**), morphological variations for leaf architecture and size were observed in amphihaploid F_1_ plants (**Figures 2D–F**) and S_1_ generation (**Figures 2G, H**). In **Figures 3A–K**, a clear difference was observed for leaf tenderness in amphihaploid plants (**Figures 3D–G**) and robust and firm leaves in S_1_ plants (**Figures 3H–K**), which resembles the natural *B. juncea* cultivars (**Figure 3C**). Furthermore, the morphology of mature plants in S_2_ generation (**Supplementary Figure S1**) clearly indicates that the resynthesized plants differ morphologically from both parental lines. The plants in the S_2_ generation exhibited significant variability in terms of plant height, number of primary branches, seeds per silique, etc., and were found to be more similar to the *B. juncea* cultivar. This indicates that the process of resynthesis might have led to some novel genetic combinations.

**Table 2:** List of resynthesized *B. juncea* (RBJ) lines developed using different accessions of parental diploid species.

S. No.	RBJ lines	Cross Details
1	RBJ 102	Rapa12 × Nigra tall
2	RBJ 104	Rapa12 × Nigra 2
3	RBJ 106	Rapa12 × IC 338498
4	RBJ 119, RBJ 120	Pusa Gold × IC 328460
5	RBJ 122, RBJ 126	Pusa Gold × IC 341132
6	RBJ 128, RBJ129, RBJ 131, RBJ 132	NRCPB rapa 8 × Nigra tall
7	RBJ 135	NRCPB rapa 8 × IC 338724
8	RBJ 137	NRCPB rapa 8 × IC 341132
9	RBJ 141 to RBJ 143, RBJ 147 to RBJ 152	NRCPB rapa 8 × IC 393266
10	RBJ 156, RBJ 159	NRCPB rapa 8× IC 399882
11	RBJ 163, 166, 167, 170, 174, 175, 179	(NRCPB rapa 8 × Rapa12) × Nigra 2
12	RBJ 186, RBJ 188	(Rapa12 × NRCPB rapa 8) × Nigra 2

### 3.2 F_1_ true hybrids were fertile after chromosome doubling

**Figure 4:**
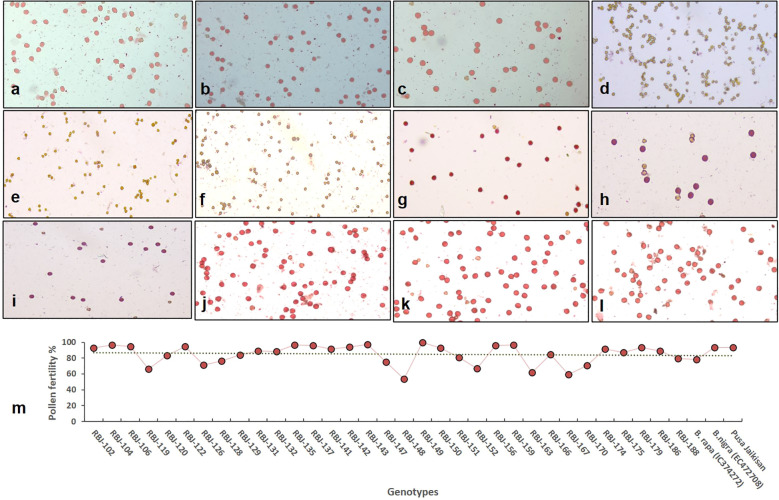
Pollen viability in **(A)** NRCPB rapa 8 (female parent), **(B)** EC472708 (*B. nigra*; male parent) and **(C)** Pusa Jaikisan (cultivar); **(D–F)** sterility in F_1_ generation and **(G–I)** fertility in S_1_ and **(J–L)** S_2_ generation of RBJ 159, RBJ 174, RBJ 179, respectively. **(M)** Graph representing the fertility of synthetic *B*. *juncea* lines in S_2_ generation along with parental diploid species viz., *B*. *rapa* (IC0623820) and *B*. *nigra* (EC472708), and *B*. *juncea* (PJK) cultivar.

The validation of hybridity in the developed amphihaploid was conducted utilizing a comprehensive set of simple sequence repeat (SSR) primers specific to the genomes involved. A total of 14 SSR primers targeting the A-genome, 18 targeting the B-genome, and 29 targeting the AB-genome were employed. The parental polymorphism was done, and the polymorphic primers were used for the hybridity confirmation. The analysis of amplification patterns obtained from these primers confirmed the hybrid nature of the developed amphihaploid. The presence of characteristic bands corresponding to the A, B, and AB genomes further substantiated the successful hybridization process (**Supplementary Figure S2**). The list of SSR primers used and hybridity assessed for each resynthesized line is given in **Supplementary Tables S2**, **S3**. **Figures 4A–C** show pollen viability of both diploid parents and the control. Pollen sterility in F_1_ hybrids was evident (nearing 100%) at the amphihaploid stage (**Figures 4D–F**), and fertility was reinstated in the S_1_ generation (**Figures 4G–I**) due to chromosome doubling with over 50% pollen stainability. Upon reaching the S_2_ generation, the synthesized lines displayed a discernible range of pollen fertility, spanning from 53% to nearly 100%, thereby culminating in an average of approximately 85% (**Figures 4J–M**).

**Figure 5:**
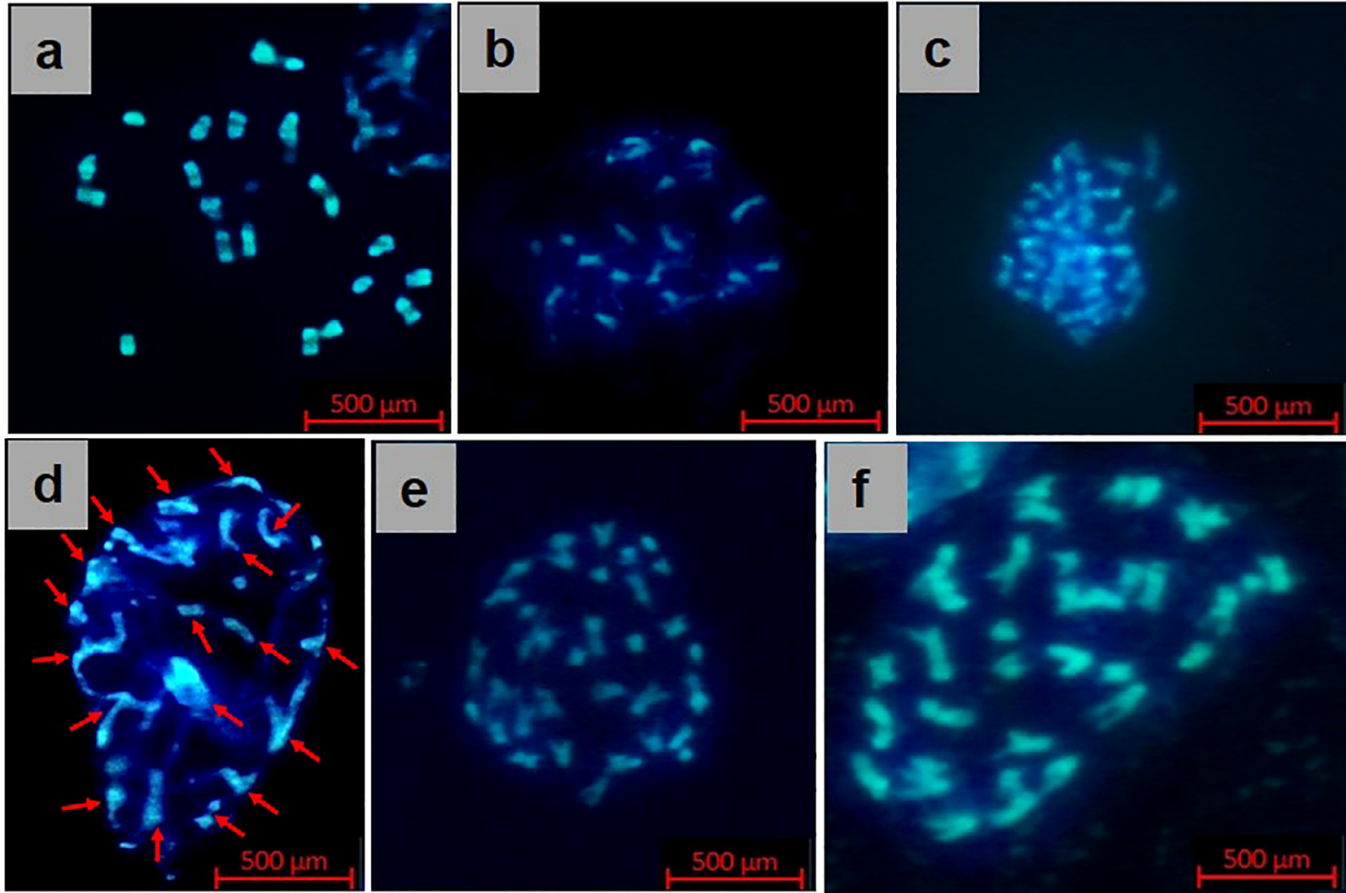
Cytogenetics (mitosis) of **(A)** *B*. *rapa* (female parent), **(B)** *B*. *nigra* (male parent), **(C)** Pusa Jaikisan (cultivar), **(D)** RBJ 122 (F_1_ generation), **(E)** RBJ 122 (S_2_ generation), and **(F)** RBJ 132 (S_2_ generation) of synthesized *B*. *juncea* lines.

The confirmed 33 true hybrids were assayed for chromosome number via mitotic configurations at the F_1_ stage, revealing 18 chromosomes under the microscope (**Figure 5D**), and the mitotic analysis of diploid parents, *B. rapa* and *B. nigra*, exhibited 20 and 16 chromosomes, respectively (**Figures 5A, B**). Thirty-six chromosomes were clearly visible in synthetic *B. juncea* lines (S_2_ generation), which was similar to the *B. juncea* cultivar Pusa Jaikisan (**Figures 5C, E, F**). The conducted cytogenetic studies established the successful development of synthetic *B. juncea* lines.

### 3.3 Significant correlation observed between different morphological traits

The genetic variation among the resynthesized genotypes with diploid parents and Indian mustard cultivars was evaluated through Analysis of Variance (ANOVA) for various phenotypic traits (**Table 3**). The results highlight significant contributions from various factors in the study. The genotypes, traits and their interactions displayed significant variations of 2.16%, 89.61% and 7.90% respectively at P > 0.0001.

**Table 3:** Analysis of variance showing mean square values and level of significance for agro-morphological traits of RBJ lines.

ANOVA	SS	df	MS	% of total variation	F	P value
Interaction	429365	376	1142	7.905	57.01	P<0.0001
Genotypes	117572	47	2502	2.164	124.9	P<0.0001
Traits	4867583	8	608448	89.61	30377	P<0.0001
Residual	17306	864	20.03			

**Figure 6:**
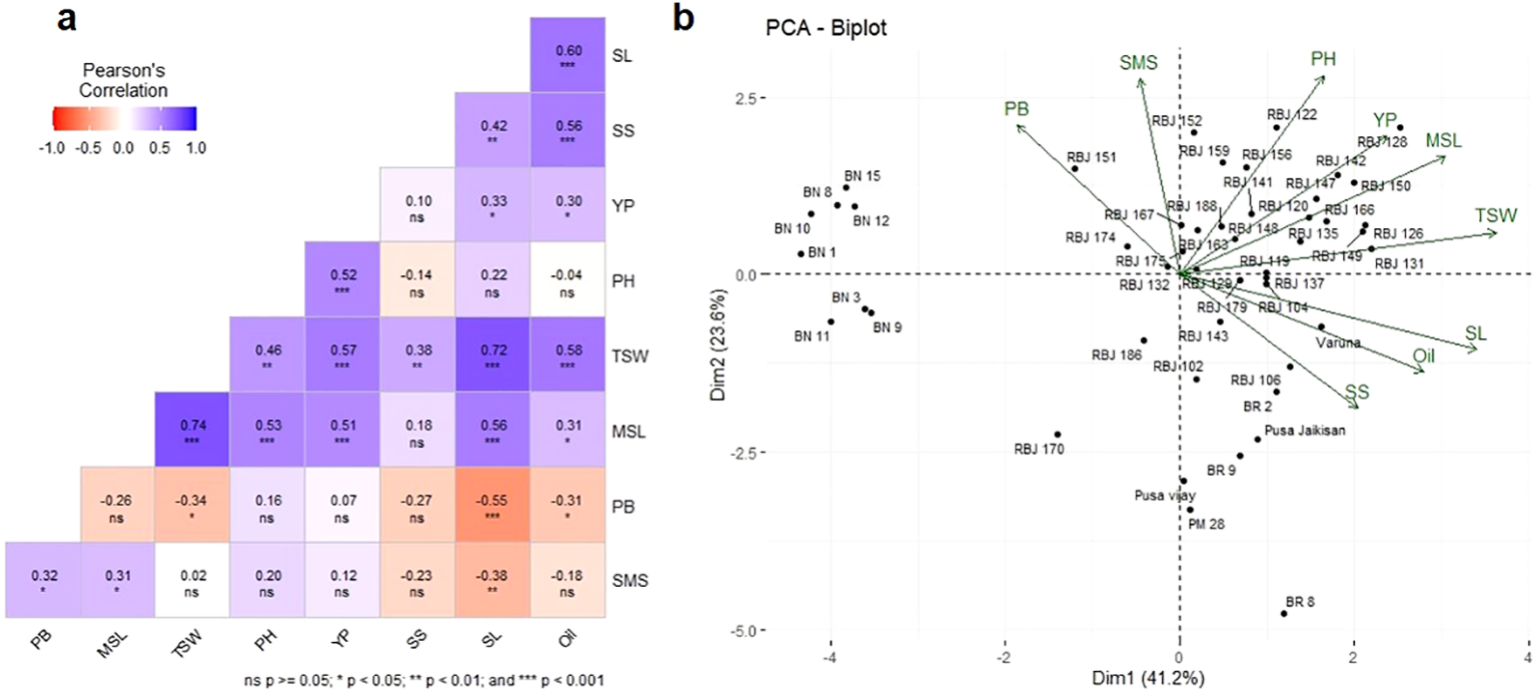
**(A)** Phenotypic correlation coefficients for evaluated morphological traits and oil content of RBJ lines. PH, Plant Height; MSL, Main Shoot Length; SL, Silique Length; SMS, Number of Siliques on Main Shoot; PB, Number of Primary Branches; SS, Seeds Per Silique; OC, Oil Content; and YP, Yield Per Plant; TSW, Thousand Seed Weight, **(B)** The biplot illustrating Principal Coordinate analysis of different morphological traits.

The correlation between different agro-morphological traits was analyzed using Pearson’s correlation coefficient, and the results are presented in **Figure 6A**. Several significant correlations were observed among the traits. Firstly, YP exhibited positive correlations with PH (r = 0.52, p < 0.001), MSL (r = 0.51, p < 0.001), and TSW (r = 0.57, p < 0.001), indicating potential associations between yield-related parameters. TSW also demonstrated strong positive correlations with PH (r = 0.46, p < 0.01), MSL (r = 0.74, p < 0.001), SL (r = 0.72, p < 0.001), SS (r =0.38, p < 0.01) and oil content (r = 0.58, p < 0.001) suggesting their potential influence on seed weight. This suggests that taller plants with longer main shoots and siliqua length tend to have higher yields. Additionally, oil content showed a positive association with SL (r = 0.60, p < 0.001) and SS (r = 0.56, p < 0.001), indicating their potential contribution to oil accumulation. This indicates that longer main shoots and siliqua length are associated with higher oil content. PH and MSL are positively correlated with r = 0.53 at p < 0.001. The weak negative correlations observed between oil content and PH (r = -0.04, p = ns) and SMS (r = -0.18, p = ns) are found to be non-significant in our study. This may imply that either there is no correlation between these two variables or this may be a random association. The positive correlation between oil content and YP (r = 0.30, p < 0.05) suggests that higher seed yield may be associated with increased oil accumulation. Oil content and YP may not be directly associated with each other but may be dependent on a complex agronomic trait, seed size or TSW which is positively correlated with both YP (r= 0.57, p< 0.001) and oil content (r= 0.58, p< 0.001). Oil content is an economically important trait and, lines with a higher oil content can thus be selected by opting for lines with higher TSW or YP. These findings provide insights into the interdependence of these traits and can guide future breeding and selection strategies to enhance specific desirable traits in Indian mustard cultivars.

Principal component analysis (PCA) (**Figure 6B**; **Supplementary Table S4**) was computed to show each trait’s contribution to the overall morphological variations. Categorized under various clusters, along with their associated morphological traits, the PCs represent axes of variation that capture the morphological diversity within the genotypes. The importance of each PC is measured by its standard deviation, percentage of variance, and cumulative proportion of variance. These statistics indicate how much each PC contributes to the overall morphological diversity captured by the entire set of PCs. The cumulative proportion of variance demonstrates that the first four PCs contribute more than 80% to the total variance, with PC1 alone accounting for about 41.2% of the total variance. It highlights the significance of specific PCs in explaining variations in morphological traits and underscores the potential implications for crop enhancement and breeding strategies.

### 3.4 Molecular and morphological diversity reflected uniqueness in the resynthesized lines of *B. juncea*

**Figure 7:**
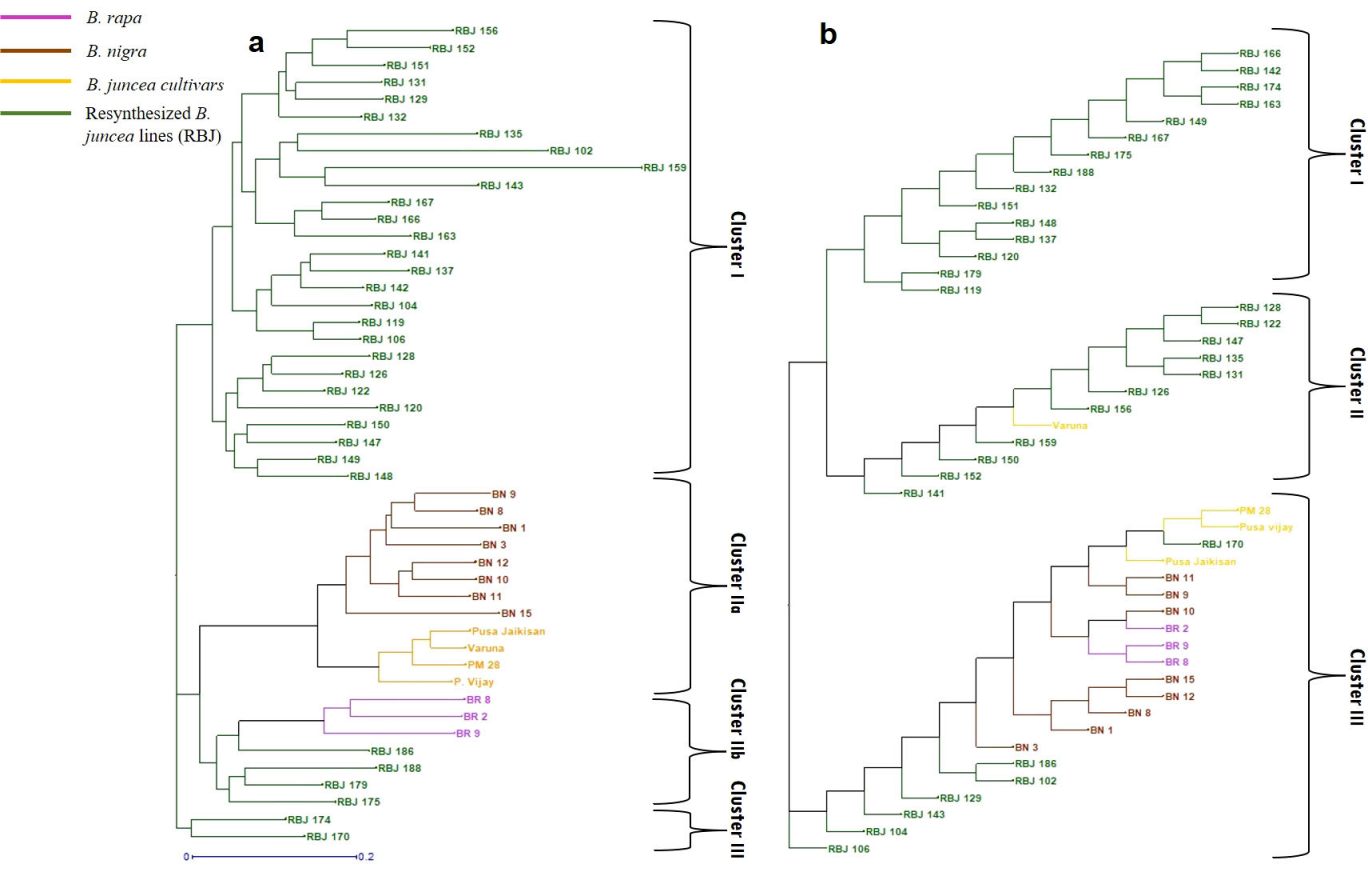
Diversity analysis based on **(A)** polymorphism generated by SSR markers and **(B)** morphological parameters. Abbreviations used: BR 2- Rapa 12, BR 8- NRCPB rapa 8, BR 9- Pusa gold, BN 1- Nigra tall, BN 3- EC426390, BN 8- IC 338498, BN 9- IC 338724, BN 10- IC 341132, BN 11- IC 393266, BN 12- IC 399882, BN 15- IC 328460.

The cluster analysis (**Figure 7A**) based on SSR markers showed that the RBJ lines were effectively distributed across all three discrete clusters. Within cluster IIb, a subset of resynthesized lines (specifically, RBJ 175, RBJ 179, RBJ 186, and RBJ 188) demonstrated close affinity with genotypes of parent *B. rapa*. This observation underscores a genomic-level congruence among these resynthesized lines and the *B. rapa* genotypes. Notably, the RBJ lines underwent subsequent sub-clustering, revealing the emergence of genomic-level diversity as a result of the resynthesis process. Moreover, the composition of cluster IIa included *B. juncea* cultivars exhibiting a more proximate phylogenetic alignment with *B. nigra* accessions. This proximity is likely attributed to historical processes of natural or artificial selection over extended timeframes.

The outcomes of the morphological cluster analysis also unveiled the presence of three prominent clusters (**Figure 7B**). The clusters I and II encompassed a substantial proportion of the synthetic *B. juncea* lines, along with the cultivar Varuna. The third cluster comprised *B. nigra* and *B. rapa* genotypes. In this cluster, a notable proximity was observed between the morphology of RBJ 170 and the cultivars Pusa Jaikisan, Pusa Vijay, and PM 28. Additionally, this cluster highlighted the close morphological proximity of RBJ 102 and RBJ 186 to the Nigra 2 genotype.

### 3.5 Population structure validates the results of genetic diversity analysis

**Figure 8:**
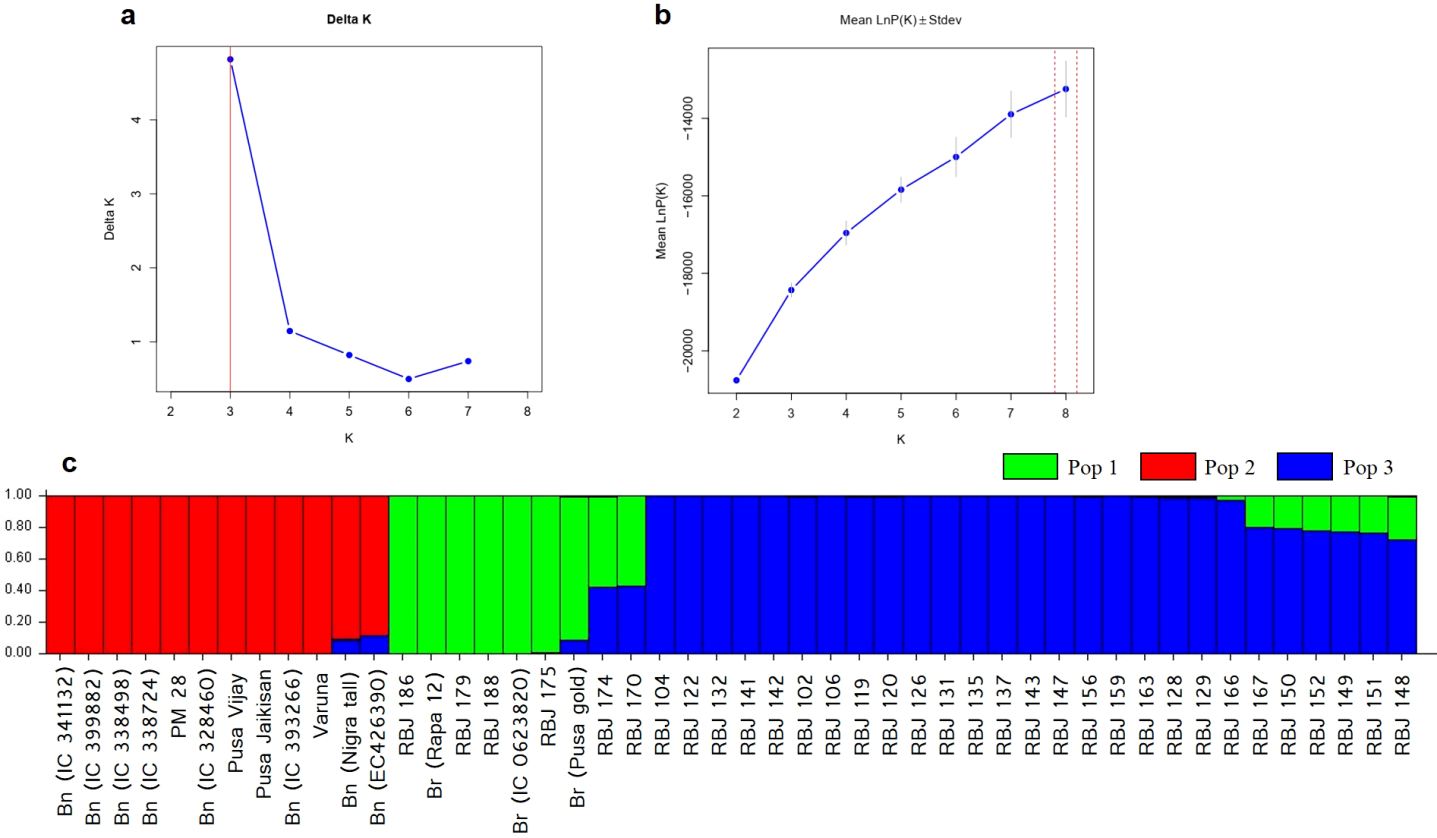
Population structure analysis **(A)** Delta *K* (Δ*K*) plot for varying *K* populations, **(B)** LnP D plot, and **(C)** Estimated population structure of Brassica genotypes used in the study at *K*=2. Br and Bn denote different accessions of *B*. *rapa* and *B*. *nigra*, i.e., parental lines.

The population structure of the *Brassica* genotypes was analyzed using STRUCTURE software. The optimal *K* value was determined by plotting the values of ΔK against the number of clusters (**Figure 8A**; **Supplementary Table S5**) that show the highest Δ*K* value at *K*=3. This indicates that the genotypes used in the study are divided into three subpopulations (pop 1, pop 2 and pop 3). The estimated Ln probability of data was -18391.7 (**Figure 8B**) with mean ln likelihood, variance of ln likelihood, and mean alpha value at -18118.9, 545.6, and 0.0381, respectively. **Table 4** summarizes the overall proportion of membership (inferred clusters), mean fixation index (Fst), divergence among the two subpopulations, and number of genotypes in each population. The genotypes were assigned to either of the populations based on the Q values from both clusters. The inferred ancestry of all individuals can be seen in **Figure 8C**; **Supplementary Table S5**.

**Table 4:** The STRUCTURE results of Brassica accessions for Fst, expected heterozygosity and no. of genotypes in each population (major).

Population	Inferred Clusters	Mean Fst	Expected Heterozygosity	No. of genotypes
Pop 1	0.246	0.5089	0.2012	9
Pop 2	0.197	0.4399	0.1995	12
Pop 3	0.557	0.6086	0.1372	27

### 3.6 High-yielding lines were observed in resynthesized lines

**Figure 9:**
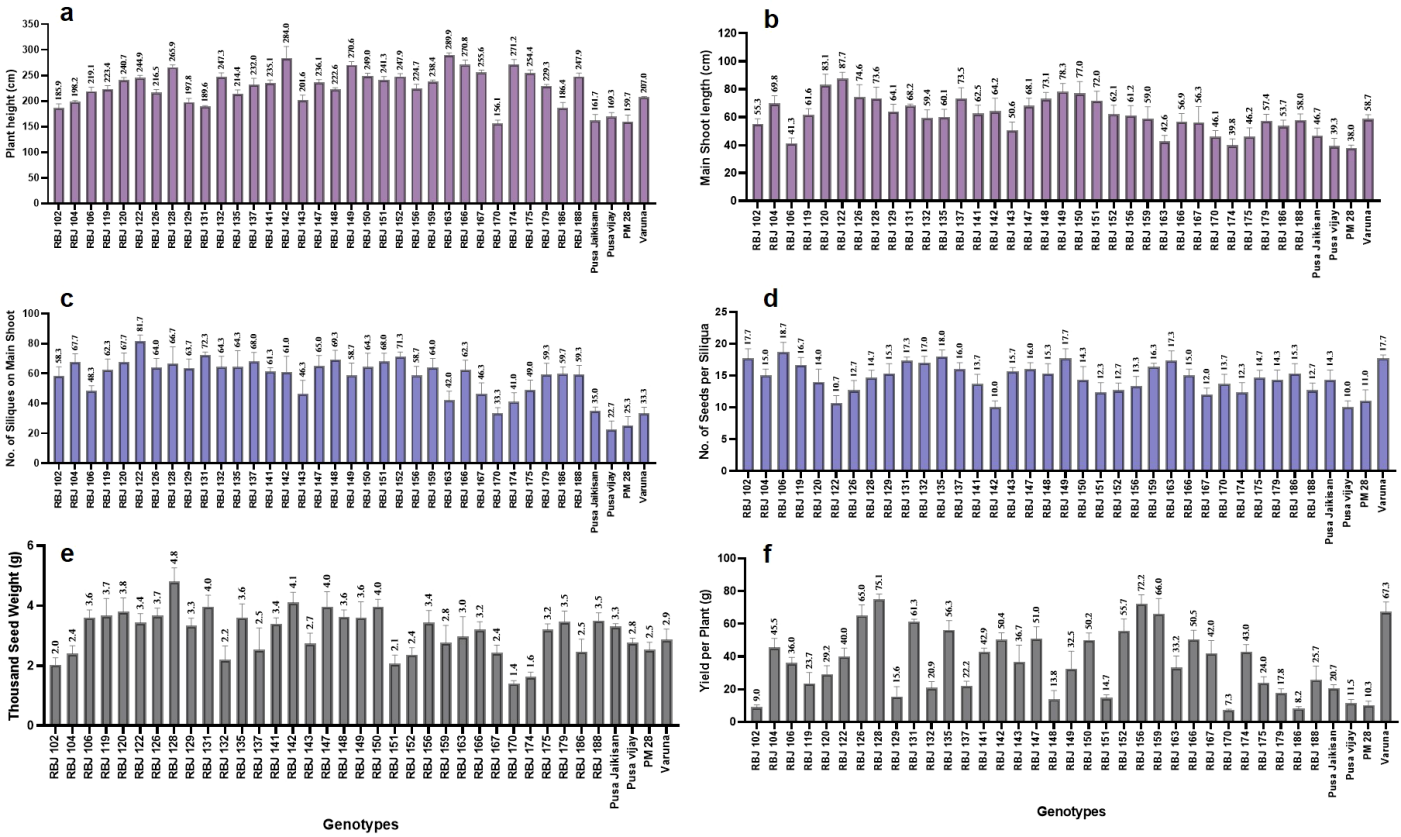
The performance of synthetic *B*. *juncea* lines (RBJ) in comparison to *B*. *juncea* cultivars for morpho-physiological traits viz., **(A)** plant height, **(B)** main shoot length, **(C)** no. of siliques on main shoot, **(D)** no. of seeds per siliqua, **(E)** thousand seed weight and **(F)** yield per plant under field conditions. Error bars: +/- 2 SE.

A clear overview of variations observed in the developed RBJ lines is presented in **Figure 9**. The synthetic lines are seen to perform better than the four *B. juncea* cultivars in terms of plant height, main shoot length, no. of siliqua on main shoot, no. of seeds per siliqua, thousand seed weight and yield per plant. RBJ 163 and RBJ 142 recorded the highest plant height of 290 cm and 284 cm, while PM 28 was the shortest at 159.7 cm. RBJ 122 has the longest main shoot length (87.7 cm) with a higher number of siliquae on the main shoot, i.e., 81.7. RBJ 106 (18.7) and RBJ 135 (18) have more seeds per siliqua followed by RBJ 149, RBJ 102 and Varuna having the same mean of 17.7. RBJ 128 observed the highest thousand seed weight (4.8) as well as yield per plant (75.1 g), while RBJ 170 has the lowest values for both traits (1.40 and 7.3 g, respectively). Yield and TSW for *B. juncea* cultivars are as follows: PJK (20.7 g, 3.3 g), P.vijay (11.5 g, 2.8 g), PM 28 (10.3 g, 2.5 g) and Varuna (67.3 g, 2.9 g).

## 4 Discussion

Due to their limited genetic diversity and narrow gene pool, oilseed Brassica, especially *B. juncea*, are extremely susceptible and non-resilient to environmental influences. Resynthesizing *B. juncea* from the existing diploid progenitor species can enhance genetic diversity, even if initial yields are lower (Gupta et al., 2015; Seyis et al., 2003; Srivastava et al., 2004). Our objective encompassed the reconstitution of *B. juncea* with the intent of enhancing genetic diversity, thereby facilitating the comprehensive evaluation of these variants across a spectrum of morphogenetic parameters. Also, the early generation characterization will help breeders to utilize the traits of interest in a time-efficient manner. As per the available data, this is the first successful attempt where the self-incompatible *B. rapa* var. toria was involved in the synthesis of *B. juncea* by deploying *B. rapa* var. yellow sarson as a bridge. It has been reported that the seed set is very low when toria is used as a female parent (Srinivasachar, 1964; Prakash, 1973). The resynthesized lines were validated through the meticulous study of mitotic events and the confirmation of hybridity using molecular markers within the F_1_ generation. Earlier researchers also reported such attempts for artificial synthesis of *B. juncea* (Bansal et al., 2009; Bhat and Sarla, 2004; Srivastava et al., 2004; Yadav et al., 2009; Hasan and Rahman, 2018) and *B. napus* (Zhang et al., 2004; Hilgert-Delgado et al., 2015; Chatterjee et al., 2016) using their progenitor species. Interestingly, crosses attempted using *B. nigra* as a female parent were unsuccessful and did not bear any seeds. Prior investigations conducted by Bhat and Sarla (2004) concluded that if *B. nigra* is used as a female parent in the synthesis of *B. juncea*, it is necessary to use tissue culture interventions to overcome post-fertilization barriers.

We observed an intermediate phenotype of the newly synthesized *B. juncea* lines. Consistent with our observations, prior work has demonstrated the development of interspecific hybrids between *B. juncea* and autotetraploid *B. fruticulosa*, resulting in intermediate phenotypes (Song et al., 2018). They reported that the size and shape of the leaves of F_1_ hybrids were closer to *B. juncea*, the female parent. Recently, fertile allohexaploid *Brassica* hybrids were developed from the crosses between *B. oleracea* and *B. juncea* (Mwathi et al., 2020). Similar to their parents, the flowers in the hybrid plants were yellow; however, the leaf morphology was intermediate between the two parents. There are allotetraploid species like *B. juncea* (Olsson, 1960; Prakash et al., 1984; Bansal et al., 2009; 2012), *B. carinata* (Prakash et al., 1984), and *B. napus* (Seyis et al., 2003) in which the increased heterozygosity attained after intergenomic crossing-over show intermediate effect on phenotypes in both resynthesized and natural types. Cytological analysis of F_1_ hybrids (n=18) confirmed hybridity and parental homeology, aligned with the results reported by Kumar et al. (2018). In contrast, our study validated amphihaploid hybridity via SSR primers for A, B, and AB genomes. Mitotic analysis in S_1_ confirmed 36 chromosomes, and cytogenetics affirmed synthetic *B. juncea* (2n=36), similar to Pusa Jaikisan.

Genomic studies on *Brassica* crops show that the current *Brassica* diploids originally came from ancient polyploids. These polyploids underwent a natural diploidization process to become functional diploids. This diploidization process involves genetic exchanges, genome restructuring, the development of new functions, modular organization, and gene silencing within a shared nucleus. This concept was first highlighted by Warwick and Black (1991), further discussed by Lagercrantz (1998), and explored by Lysak et al. (2005). Additionally, the technique of derived amphiploidy, as introduced by Banga and Kaur (2009), is rooted in the natural notion of cyclic polyploidy in which a genome that has undergone diploidization may recurrently participate in multiple rounds of genome merging, duplication, and diploidization. Wang et al. (2021) also discussed about this concept in his review and how genome downsizing occurs and is selectively favored. The concept of whole genome duplication and post-polyploidy genome divergence is discussed by many researchers (Leitch and Bennett, 2004; Wendel, 2015; Zenil-Ferguson et al., 2016; Pellicer et al., 2018). This mechanism contributes to the intricate evolutionary processes observed in *Brassica* species. This can be observed in the SSR-based clustering analysis, where the resynthesized genotypes were grouped into a separate cluster, whereby we found that RBJ lines were more closely related to *B. rapa*. One might argue this to be the case when using *B. rapa* as a female parent. However, it is to be emphasized that the cytoplasm does not play a significant role in genetic clustering, and the resynthesized types with *B. nigra* cytoplasm may also show closer proximity with *B. rapa* in terms of genetic relatedness (Bansal et al., 2009). The resynthesized lines having one or both common diploid parents may not always fall in the same group. However, most of the resynthesized lines generated through three-way crosses involving a hybrid of *B. rapa* var. toria, *B. rapa* var. yellow sarson, and *B. nigra* (RBJ 170, RBJ 174, RBJ 175, RBJ 179, RBJ 186, RBJ 188) were found to be in the same cluster and were distantly placed from other resynthesized lines, thus indicating the contribution of *B. rapa* var. toria in generating this genetic diversity. The significance of a three-way cross in creating a gene pool with high genetic variance is well demonstrated in this study. The PCA and clustering analysis for agronomic traits also revealed the successful synthesis of *B. juncea* lines, which were highly diverse but similar to natural cultivars of *B. juncea*. Out of 33 RBJ lines, 11 lines were morphologically more similar to the *B. juncea* cultivar Varuna. Surprisingly, RBJ 170 was most closely related to PJK, Pusa Vijay and PM 28 in terms of agronomic traits.

Pandey et al. (2021) reported that the differing alleles among clusters can help in detecting the principal differences and thus can lead to the use of these genotypes for breeding successfully. According to Meirmans (2015), there is a chance of uncertainty in inferring *K* and hence, correlating these results with PCA and the phylogenetic tree obtained by DARwin is extremely helpful. The population genetics results determined by STRUCTURE at *K*=3 (**Figure 8C**) and DARwin (**Figure 7A**) perfectly align forming three clusters. Here also, based on the Q values, resynthesized lines obtained by three-way crosses are closer to the *B. rapa* accessions, as in **Figure 7A**. The studies conducted by Giri et al. (2017); Zhao et al. (2018); Zhou et al. (2018), and Luo et al. (2019) also reported similar observations.

The prior studies done by Scannell et al. (2007) suggests that the age/generation of the polyploids also play an important role in genome size, DNA loss and recombination frequencies as reviewed by Wang et al. (2021). Due to higher recombination frequency in early-generation polyploids (compared to stable and advanced polyploids) (Yant et al., 2013; Lloyd and Bomblies, 2016), these polyploids have a higher chromosomal and genetic diversity. Indeed, it has been hypothesized that a positive feedback loop exists, whereby homeologous recombination in young allopolyploids causes depletion in DNA mismatch repair proteins, which enhances aberrant recombination and DNA loss, leading to even more homeologous recombination in future generations (Comai, 2000). Also, it has been seen that DNA loss is more in early-stage polyploid as reported in *Phlox drummondii*, in which there was a decrease in genome size by one-fourth (Raina et al., 1994). This might offer a selective advantage. Selection from this diversity could favor variants with smaller GS (Wang et al., 2021).

## 5 Conclusion

We report the successful synthesis of allotetraploid *B. juncea* lines using the two diploid progenitor species. For the development of the synthetic amphidiploid *B. juncea* lines, *B. rapa* and *B. nigra* were crossed to make synthetic amphihaploids (AB, chromosome number 18), followed by the chromosome doubling and further selfing in the subsequent generations. Due to continuous selfing and selection in the subsequent generations, the chromosomal rearrangement and inter-genomic interactions for the stability and homozygosity will prevail leading to draining of the genetic variability in the synthetic *B. juncea* lines over the generations. Thus, characterizing and utilizing these new *B. juncea* lines is a promising strategy for harnessing maximum parental genomic diversity to improve Indian mustard. Involving early-stage synthetic lines in the breeding program can save time as the generations used for the advancement and stability of these lines can be utilized in the varietal development. Assessing the potential of these lines/traits for integration into *B. juncea* enhancement initiatives, particularly targeting seed and oil yield, shall open new crop breeding opportunities.

## Data Availability

The original contributions presented in the study are included in the article/**Supplementary Material**. Further inquiries can be directed to the corresponding authors.

## Supplementary material

Supplementary Figure 1 Morphology of plants in the field. NRCPB rapa 8 (P1); Nigra Dwarf (P2); Pusa Jaikisan (cultivar) (PJK) and S_2_ generation of resynthesized *B. juncea* lines (RBJ 104, RBJ 106, RBJ 131, RBJ 137, RBJ 141, RBJ 143, RBJ 170 and RBJ 188).

Supplementary Figure 2 Representative image of hybridity confirmation of resynthesized amphihaploid hybrids (33) at F_1_ generation using SSR primer UGM 632 on 2.5% agarose gel and 100bp ladder (L).
