# Supplementary figures and images for: Insights into early generation synthetic amphidiploid Brassica juncea: a strategy to harness maximum parental genomic diversity for improving Indian mustard
Source: Front Plant Sci. 2025 Feb 13;16:1493618. doi: 10.3389/fpls.2025.1493618 (PMC11865204; doi:10.3389/fpls.2025.1493618)

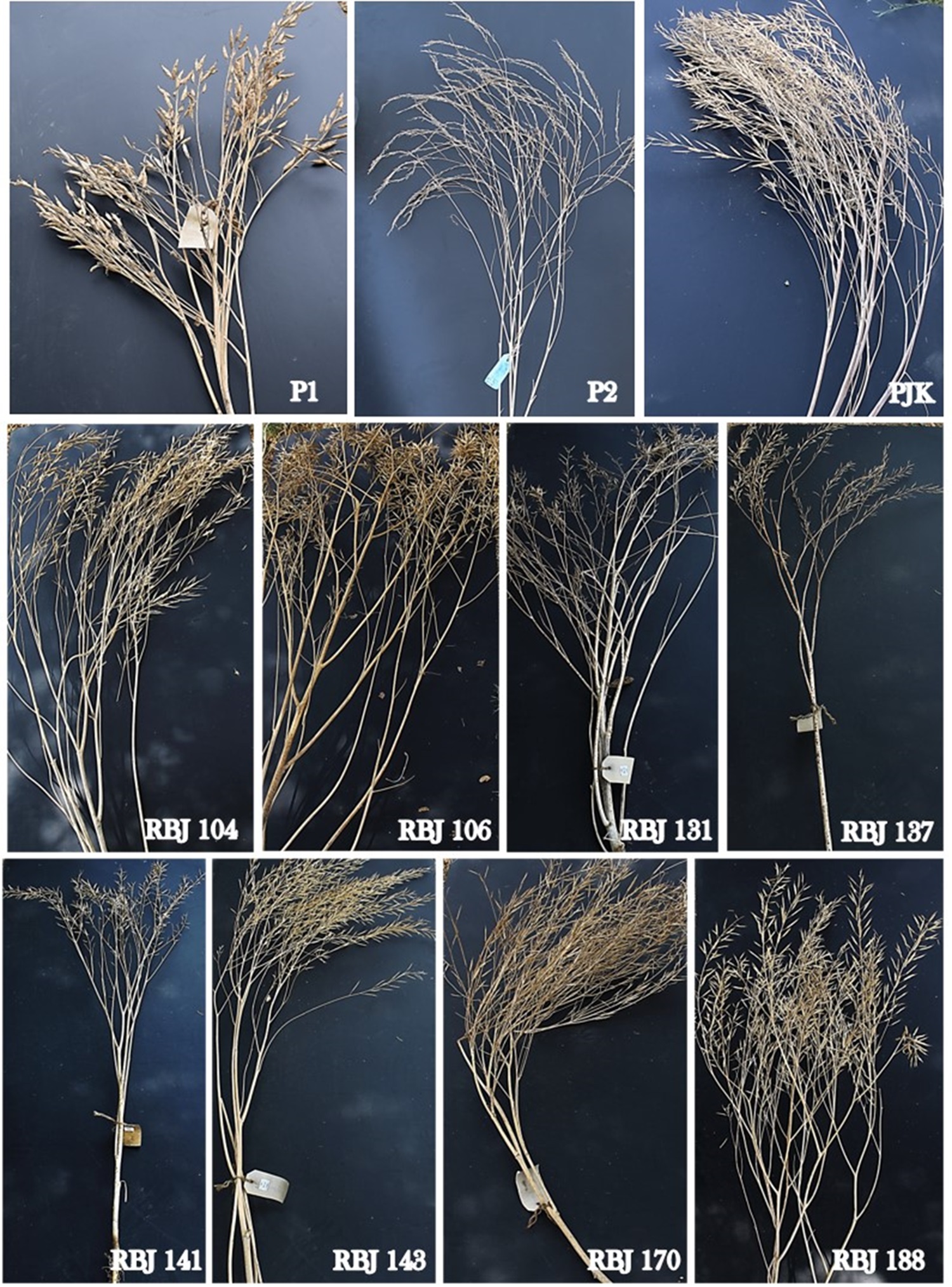

Supplement: Supplementary Figure 1 — Morphology of plants in the field. NRCPB rapa 8 (P1); Nigra Dwarf (P2); Pusa Jaikisan (cultivar) (PJK) and S2 generation of resynthesized B. juncea lines (RBJ 104, RBJ 106, RBJ 131, RBJ 137, RBJ 141, RBJ 143, RBJ 170 and RBJ 188). [file Image1.jpeg]

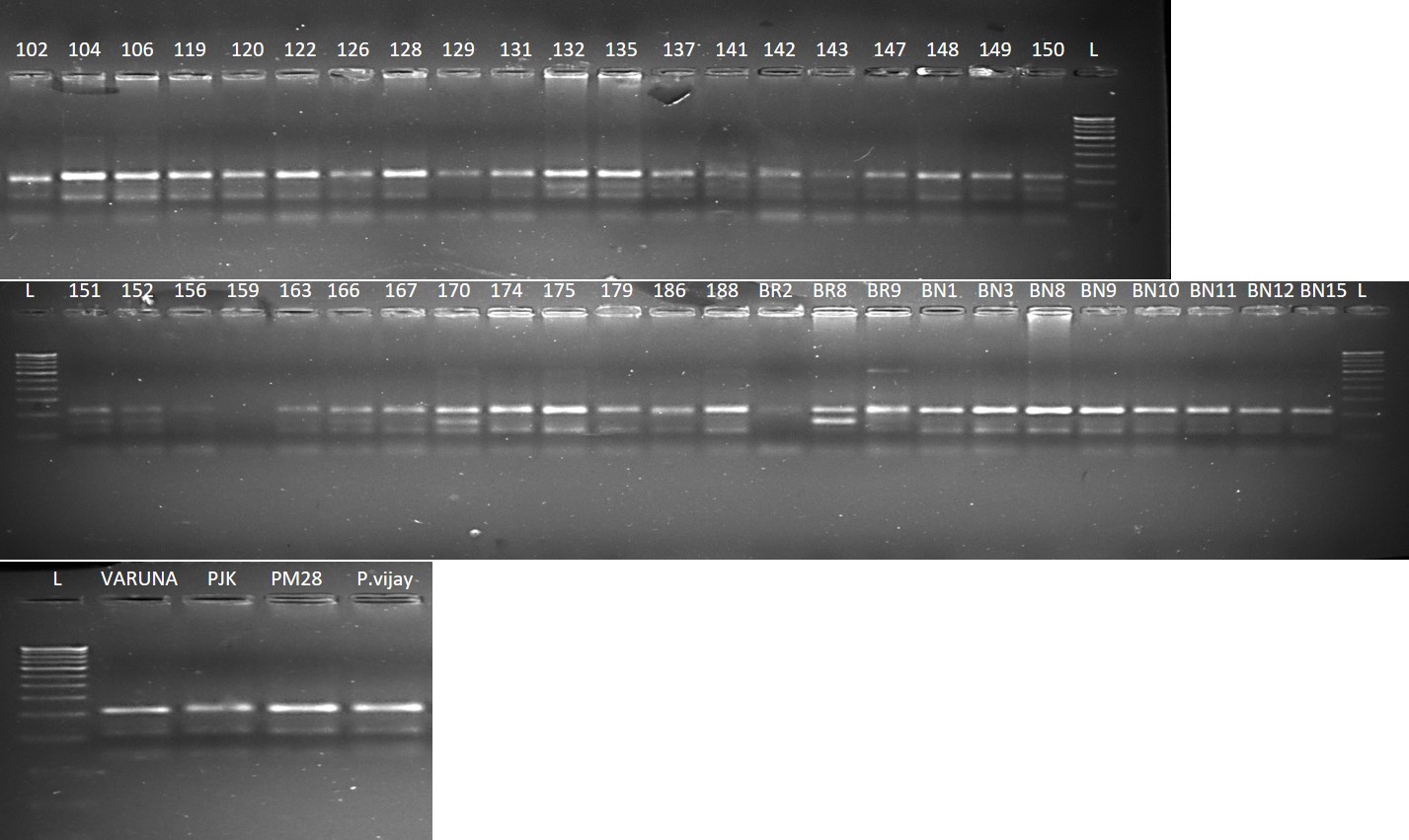

Supplement: Supplementary Figure 2 — Representative image of hybridity confirmation of resynthesized amphihaploid hybrids (33) at F1 generation using SSR primer UGM 632 on 2.5% agarose gel and 100bp ladder (L). [file Image2.jpeg]
