# Supplementary material for: Insights into early generation synthetic amphidiploid Brassica juncea: a strategy to harness maximum parental genomic diversity for improving Indian mustard
Source: Front Plant Sci. 2025 Feb 13;16:1493618. doi: 10.3389/fpls.2025.1493618 (PMC11865204; doi:10.3389/fpls.2025.1493618)
Supplement: Supplementary file 3 [file Table1.docx]

Supplementary Material

**Journal:** Frontiers in Plant Science

**Title:** Insights into early generation synthetic amphidiploid *Brassica juncea*: A strategy to harness the maximum parental genomic diversity for improving Indian mustard

Author(s): Pooja Garg^1,2†^, Shikha Tripathi^1,3†^, Anamika Kashyap^1,4^, A. Anil Kumar^5^, Sujata Kumari^1^, Mandeep Singh^6,7^, Ranjeet Kushwaha^1^, Shiv Shankar Sharma^1^, Jyoti Sharma^1^, Rashmi Yadav^8^, N.C. Gupta^1^, Naveen Singh^6^, Ramcharan Bhattacharya^1*^, Vinod Chhokar^2*^ and Mahesh Rao^1*^

**Author affiliation:**

^1^Indian Council of Agricultural Research (ICAR)- National Institute for Plant Biotechnology (NIPB), New Delhi-110012, India

^2^Department of Biotechnology, Guru Jambheshwar University of Science and Technology (GJUS&T), Hisar, Haryana-125001, India

^3^Department of Botany, Institute of Science, Banaras Hindu University (BHU), Varanasi, Uttar Pradesh-221005, India

^4^Veer Chandra Singh Garhwali Uttarakhand University of Horticulture & Forestry, Bharsar, Pauri

Garhwal, Uttarakhand-246123, India

^5^Crop Improvement Section, ICAR- Indian Institute of Oilseeds Research, Hyderabad (IIOR), Telangana-500030, India

^6^Division of Genetics, ICAR-Indian Agricultural Research Institute (IARI), New Delhi- 110012, India

^7^Applied Genomics Section, Bhabha Atomic Research Centre (BARC), Mumbai-400085, India

^8^Division of Germplasm Evaluation, ICAR- National Bureau of Plant Genetic Resources (NBPGR), New Delhi-110012, India

^†^ These authors contributed equally to this work

***Corresponding authors**

- Dr. Mahesh Rao, Senior Scientist, ICAR- National Institute for Plant Biotechnology, Pusa campus, New Delhi-110012, India. Email: [mraoicar@gmail.com](mailto:mraoicar@gmail.com). Ph: +918700040940
- Prof. Vinod Chhokar, Registrar, Guru Jambheshwar University of Science and Technology, Hisar, Haryana-125001, India. Email: [vinodchhokar@yahoo.com](mailto:vinodchhokar@yahoo.com). Ph: +919992793333
- Dr. Ramcharan Bhattacharya, Principal Scientist, ICAR- National Institute for Plant Biotechnology, Pusa campus, New Delhi-110012, India. Email: rcbhattacharya1@gmail.com. Ph: +919868357986

**Supplementary Table S1.** Different cross combinations of *B. rapa* and *B. nigra* attempted in generation of resynthetic *B. juncea.*

| S. No. | Cross Details | No. of Buds pollinated | No. of Seeds obtained |
| --- | --- | --- | --- |
| *Brassica rapa* X *Brassica nigra* | | | |
|  | Rapa 12 x Bn (nigra tall) | 148 | 291 |
|  | Rapa 12 x Bn (EC-472708) | 156 | 67 |
|  | Rapa 12 x Bn (IC-338498) | 119 | 9 |
|  | Rapa 12 x Bn (IC-341132) | 119 | 0 |
|  | Rapa 12 x Bn (IC-393266) | 48 | 0 |
|  | Rapa 12 x Bn (IC-328460) | 158 | 0 |
|  | Pusa Gold x Bn (nigra tall) | 115 | 0 |
|  | Pusa Gold x Bn (IC-338498) | 303 | 0 |
|  | Pusa Gold x Bn (IC-338724) | 297 | 0 |
|  | Pusa Gold x Bn (IC-393266) | 117 | 0 |
|  | Pusa Gold x Bn (IC-341132) | 309 | 30 |
|  | Pusa Gold x Bn (IC-399882) | 106 | 0 |
|  | Pusa Gold x Bn (IC-328460) | 106 | 65 |
|  | NRCPB Rapa 8 x Bn (nigra tall) | 143 | 221 |
|  | NRCPB Rapa 8 x Bn (EC-472708) | 136 | 0 |
|  | NRCPB Rapa 8 x Bn (IC-338498) | 154 | 0 |
|  | NRCPB Rapa 8 x Bn (IC-338724) | 129 | 280 |
|  | NRCPB Rapa 8 x Bn (IC-341132) | 161 | 48 |
|  | NRCPB Rapa 8 x Bn (IC-393266) | 117 | 316 |
|  | NRCPB Rapa 8 x Bn (IC-399882) | 134 | 126 |
|  | NRCPB Rapa 8 x Bn (IC-328460) | 152 | 0 |
| *Brassica rapa X Brassica nigra* | | | |
|  | Bn (IC-399882) × Pusa Gold | 87 | 0 |
|  | Bn (IC-341132) x Pusa Gold | 52 | 0 |
|  | Bn (IC-341132) x NRCPB Rapa 8 | 46 | 0 |
|  | Bn (IC-399882) x NRCPB Rapa 8 | 47 | 0 |
|  | Bn (IC 328460) x NRCPB Rapa8 | 53 | 0 |
| Three-way Cross | | | |
| 27. | [NRCPB Rapa 8 x Br (Rapa 12)] * Nigra 2 (EC 472708) |  |  |
| 28. | [Br (Rapa 12) x NRCPB Rapa 8] * Nigra 2 (EC 472708) |  |  |
