# Supplementary material for: Insights into early generation synthetic amphidiploid Brassica juncea: a strategy to harness maximum parental genomic diversity for improving Indian mustard
Source: Front Plant Sci. 2025 Feb 13;16:1493618. doi: 10.3389/fpls.2025.1493618 (PMC11865204; doi:10.3389/fpls.2025.1493618)
Supplement: Supplementary file 6 [file Table4.docx]

Supplementary Material

**Journal:** Frontiers in Plant Science

**Title:** Insights into early generation synthetic amphidiploid *Brassica juncea*: A strategy to harness the maximum parental genomic diversity for improving Indian mustard

Author(s): Pooja Garg^1,2†^, Shikha Tripathi^1,3†^, Anamika Kashyap^1,4^, A. Anil Kumar^5^, Sujata Kumari^1^, Mandeep Singh^6,7^, Ranjeet Kushwaha^1^, Shiv Shankar Sharma^1^, Jyoti Sharma^1^, Rashmi Yadav^8^, N.C. Gupta^1^, Naveen Singh^6^, Ramcharan Bhattacharya^1*^, Vinod Chhokar^2*^ and Mahesh Rao^1*^

**Author affiliation:**

^1^Indian Council of Agricultural Research (ICAR)- National Institute for Plant Biotechnology (NIPB), New Delhi-110012, India

^2^Department of Biotechnology, Guru Jambheshwar University of Science and Technology (GJUS&T), Hisar, Haryana-125001, India

^3^Department of Botany, Institute of Science, Banaras Hindu University (BHU), Varanasi, Uttar Pradesh-221005, India

^4^Veer Chandra Singh Garhwali Uttarakhand University of Horticulture & Forestry, Bharsar, Pauri

Garhwal, Uttarakhand-246123, India

^5^Crop Improvement Section, ICAR- Indian Institute of Oilseeds Research, Hyderabad (IIOR), Telangana-500030, India

^6^Division of Genetics, ICAR-Indian Agricultural Research Institute (IARI), New Delhi- 110012, India

^7^Applied Genomics Section, Bhabha Atomic Research Centre (BARC), Mumbai-400085, India

^8^Division of Germplasm Evaluation, ICAR- National Bureau of Plant Genetic Resources (NBPGR), New Delhi-110012, India

^†^ These authors contributed equally to this work

***Corresponding authors**

- Dr. Mahesh Rao, Senior Scientist, ICAR- National Institute for Plant Biotechnology, Pusa campus, New Delhi-110012, India. Email: [mraoicar@gmail.com](mailto:mraoicar@gmail.com). Ph: +918700040940
- Prof. Vinod Chhokar, Registrar, Guru Jambheshwar University of Science and Technology, Hisar, Haryana-125001, India. Email: [vinodchhokar@yahoo.com](mailto:vinodchhokar@yahoo.com). Ph: +919992793333
- Dr. Ramcharan Bhattacharya, Principal Scientist, ICAR- National Institute for Plant Biotechnology, Pusa campus, New Delhi-110012, India. Email: rcbhattacharya1@gmail.com. Ph: +919868357986

**Supplementary Table S4.** Eigenvalues and contribution values obtained from PCA analysis

**Eigenvalues**

| Component | Eigenvalue | % of variance | Cumulative % of variance |
| --- | --- | --- | --- |
| 1 | 3.707624848 | 41.19583165 | 41.19583165 |
| 2 | 2.119599247 | 23.55110275 | 64.7469344 |
| 3 | 0.928541088 | 10.3171232 | 75.0640576 |
| 4 | 0.835522556 | 9.283583961 | 84.34764156 |
| 5 | 0.47588061 | 5.287562335 | 89.6352039 |
| 6 | 0.380056251 | 4.222847233 | 93.85805113 |
| 7 | 0.251826218 | 2.798069087 | 96.65612021 |
| 8 | 0.171761453 | 1.90846059 | 98.5645808 |
| 9 | 0.129187728 | 1.435419196 | 100 |

**Contribution values**

| Component | Dim 1 | Dim 2 | Dim 3 | Dim 4 | Dim 5 |
| --- | --- | --- | --- | --- | --- |
| PH | 4.690444459 | 23.58594343 | 7.955593432 | 8.39635822 | 19.23718122 |
| MSL | 15.98219415 | 8.268025269 | 0.882767837 | 12.88924151 | 2.060492449 |
| SMS | 0.353619077 | 22.76248228 | 14.43201343 | 40.41438427 | 0.635135897 |
| PB | 5.968499843 | 13.29205282 | 21.48344614 | 21.41395443 | 2.297179434 |
| SL | 19.95118292 | 3.365859283 | 6.300851359 | 0.338880799 | 0.041099893 |
| SS | 7.1899441 | 10.66924079 | 29.4420745 | 0.075279062 | 37.11367707 |
| YP | 9.692561465 | 11.34885734 | 1.698854626 | 15.95692373 | 23.2219378 |
| TSW | 22.74853492 | 1.057434844 | 0.01581145 | 0.210930493 | 0.000710788 |
| Oil | 13.42301906 | 5.650103948 | 17.78858722 | 0.304047475 | 15.39258545 |

**Individual Contribution values for each genotype**

|  | Dim 1 | Dim 2 | Dim 3 | Dim 4 | Dim 5 |
| --- | --- | --- | --- | --- | --- |
| RBJ 102 | 0.020390416 | 2.110023297 | 0.588993888 | 1.212165287 | 0.172136626 |
| RBJ 104 | 0.557928732 | 0.01537893 | 0.008601574 | 3.30809896 | 3.063165346 |
| RBJ 106 | 0.893666096 | 1.649807859 | 0.356700776 | 0.118672031 | 0.04688043 |
| RBJ 119 | 0.550041413 | 0.000510696 | 0.296331318 | 0.38187492 | 0.802679554 |
| RBJ 120 | 1.230389369 | 0.617397614 | 1.095518062 | 5.50474382 | 0.462441527 |
| RBJ 122 | 0.678739848 | 4.2345286 | 1.271103455 | 7.079974062 | 0.001702657 |
| RBJ 126 | 2.536695895 | 0.484289025 | 0.00025048 | 0.339347092 | 8.341882211 |
| RBJ 128 | 3.579644928 | 4.194704588 | 0.034598144 | 0.413414164 | 0.701546047 |
| RBJ 129 | 0.019585525 | 0.0057144 | 0.021455949 | 1.161725171 | 1.824518132 |
| RBJ 131 | 2.700606702 | 0.128501142 | 2.637198458 | 0.712559944 | 4.313737071 |
| RBJ 132 | 0.011559 | 0.012321745 | 2.127549353 | 2.462280258 | 6.49028905 |
| RBJ 135 | 1.065746719 | 0.209181524 | 0.254050292 | 0.003839766 | 0.006308474 |
| RBJ 137 | 0.554750542 | 0.001555345 | 0.125482041 | 4.748051358 | 0.462836939 |
| RBJ 141 | 0.382529703 | 0.724405578 | 0.006675213 | 0.110228264 | 0.024844346 |
| RBJ 142 | 1.846714369 | 1.900606406 | 0.712060561 | 0.200879067 | 2.034969501 |
| RBJ 143 | 0.121112646 | 0.427344082 | 0.137253172 | 1.999339586 | 0.162671488 |
| RBJ 147 | 1.375837147 | 1.096105154 | 0.6757666 | 0.028744083 | 0.01494042 |
| RBJ 148 | 0.225781687 | 0.245982102 | 0.13530378 | 4.542023526 | 3.453464027 |
| RBJ 149 | 2.462470686 | 0.371247837 | 0.052161748 | 0.041884879 | 3.791945027 |
| RBJ 150 | 2.220059202 | 1.649886907 | 0.079741579 | 0.008158389 | 0.021330688 |
| RBJ 151 | 0.818965057 | 2.177494416 | 1.621095748 | 1.632841728 | 8.583529973 |
| RBJ 152 | 0.015428868 | 3.944192105 | 2.375081902 | 1.741040376 | 0.476672394 |
| RBJ 156 | 0.321628283 | 2.243819987 | 1.504616181 | 3.37687811 | 3.596395298 |
| RBJ 159 | 0.136590881 | 2.413736893 | 1.282419918 | 2.945660717 | 0.011085343 |
| RBJ 163 | 0.021982028 | 0.385552716 | 0.399299605 | 14.40834386 | 7.360754901 |
| RBJ 166 | 1.584474055 | 0.543366973 | 0.009007887 | 0.731434053 | 0.055395121 |
| RBJ 167 | 5.94E-05 | 0.474540882 | 1.485246245 | 3.360455733 | 0.222770121 |
| RBJ 170 | 1.11038979 | 4.958355514 | 3.790238303 | 0.001030798 | 0.141698584 |
| RBJ 174 | 0.200619888 | 0.156026586 | 3.05275977 | 9.47508502 | 1.015931501 |
| RBJ 175 | 0.000613119 | 0.104722919 | 0.955296679 | 4.818340305 | 1.08678214 |
| RBJ 179 | 0.267576632 | 0.0053091 | 0.162158612 | 0.222610885 | 0.787333128 |
| RBJ 186 | 0.096344734 | 0.836447166 | 0.875742077 | 2.567920179 | 1.589426686 |
| RBJ 188 | 0.12287383 | 0.446221631 | 1.98862911 | 0.004040767 | 1.881428919 |
| Br (Rapa 12) | 0.68756562 | 2.66611246 | 4.03077757 | 0.766931632 | 9.753576881 |
| Br (IC 0623820) | 0.796883083 | 22.39005844 | 25.19424973 | 0.005340923 | 10.56262597 |
| Br (Pusa gold) | 0.265944426 | 6.337285981 | 7.277579028 | 0.758505286 | 0.173388344 |
| Bn (Nigra tall) | 10.6068107 | 0.08213286 | 0.27268498 | 0.091391857 | 0.683903627 |
| Bn (EC426390) | 7.306892414 | 0.238717973 | 0.000704044 | 2.510331316 | 0.004366022 |
| Bn (IC 338498) | 8.642418877 | 0.951597306 | 0.031072271 | 0.989431294 | 0.022477043 |
| Bn (IC 338724) | 7.021459758 | 0.279217246 | 0.083905807 | 0.184022228 | 1.041069014 |
| Bn (IC 341132) | 10.01124829 | 0.715403778 | 1.887522909 | 0.098945707 | 0.001894609 |
| Bn (IC 393266) | 8.998955632 | 0.429700625 | 0.728382983 | 3.08221358 | 0.806220585 |
| Bn (IC 399882) | 7.779224039 | 0.89519422 | 1.812144669 | 2.751350579 | 2.453406441 |
| Bn (IC 328460) | 8.226396923 | 1.48279577 | 1.838474281 | 2.207936904 | 3.175905971 |
| Pusa Jaikisan | 0.443683412 | 5.284828512 | 1.321020909 | 0.27291918 | 1.346415231 |
| Pusa Vijay | 0.000982314 | 8.255970729 | 17.20931216 | 0.05710291 | 1.273654957 |
| PM 28 | 0.008324764 | 10.68313015 | 7.859714418 | 0.001167246 | 4.908594722 |
| Varuna | 1.47141258 | 0.538574235 | 0.33406577 | 6.558722202 | 0.789006916 |
