# Supplementary material for: Insights into early generation synthetic amphidiploid Brassica juncea: a strategy to harness maximum parental genomic diversity for improving Indian mustard
Source: Front Plant Sci. 2025 Feb 13;16:1493618. doi: 10.3389/fpls.2025.1493618 (PMC11865204; doi:10.3389/fpls.2025.1493618)
Supplement: Supplementary file 7 [file Table5.docx]

Supplementary Material

**Journal:** Frontiers in Plant Science

**Title:** Insights into early generation synthetic amphidiploid *Brassica juncea*: A strategy to harness the maximum parental genomic diversity for improving Indian mustard

Author(s): Pooja Garg^1,2†^, Shikha Tripathi^1,3†^, Anamika Kashyap^1,4^, A. Anil Kumar^5^, Sujata Kumari^1^, Mandeep Singh^6,7^, Ranjeet Kushwaha^1^, Shiv Shankar Sharma^1^, Jyoti Sharma^1^, Rashmi Yadav^8^, N.C. Gupta^1^, Naveen Singh^6^, Ramcharan Bhattacharya^1*^, Vinod Chhokar^2*^ and Mahesh Rao^1*^

**Author affiliation:**

^1^Indian Council of Agricultural Research (ICAR)- National Institute for Plant Biotechnology (NIPB), New Delhi-110012, India

^2^Department of Biotechnology, Guru Jambheshwar University of Science and Technology (GJUS&T), Hisar, Haryana-125001, India

^3^Department of Botany, Institute of Science, Banaras Hindu University (BHU), Varanasi, Uttar Pradesh-221005, India

^4^Veer Chandra Singh Garhwali Uttarakhand University of Horticulture & Forestry, Bharsar, Pauri

Garhwal, Uttarakhand-246123, India

^5^Crop Improvement Section, ICAR- Indian Institute of Oilseeds Research, Hyderabad (IIOR), Telangana-500030, India

^6^Division of Genetics, ICAR-Indian Agricultural Research Institute (IARI), New Delhi- 110012, India

^7^Applied Genomics Section, Bhabha Atomic Research Centre (BARC), Mumbai-400085, India

^8^Division of Germplasm Evaluation, ICAR- National Bureau of Plant Genetic Resources (NBPGR), New Delhi-110012, India

^†^ These authors contributed equally to this work

***Corresponding authors**

- Dr. Mahesh Rao, Senior Scientist, ICAR- National Institute for Plant Biotechnology, Pusa campus, New Delhi-110012, India. Email: [mraoicar@gmail.com](mailto:mraoicar@gmail.com). Ph: +918700040940
- Prof. Vinod Chhokar, Registrar, Guru Jambheshwar University of Science and Technology, Hisar, Haryana-125001, India. Email: [vinodchhokar@yahoo.com](mailto:vinodchhokar@yahoo.com). Ph: +919992793333
- Dr. Ramcharan Bhattacharya, Principal Scientist, ICAR- National Institute for Plant Biotechnology, Pusa campus, New Delhi-110012, India. Email: rcbhattacharya1@gmail.com. Ph: +919868357986

**Supplementary Table S5.** Delta K values as determined by the StructureSelector for varying *K* values.

| **K** | **Reps** | **Mean LnP(K)** | **Stdev LnP(K)** | **Ln'(K)** | **\|Ln''(K)\|** | **Delta K** |
| --- | --- | --- | --- | --- | --- | --- |
| 2 | 5 | -20757.22000 | 5.05688 | NA | NA | NA |
| 3 | 5 | -18429.12000 | 177.15591 | 2328.10000 | 853.82000 | 4.81960 |
| 4 | 5 | -16954.84000 | 312.18216 | 1474.28000 | 357.82000 | 1.14619 |
| 5 | 5 | -15838.38000 | 330.95132 | 1116.46000 | 272.00000 | 0.82187 |
| 6 | 5 | -14993.92000 | 513.94027 | 844.46000 | 256.12000 | 0.49835 |
| 7 | 5 | -13893.34000 | 605.73811 | 1100.58000 | 447.72000 | 0.73913 |
| 8 | 5 | -13240.48000 | 727.43617 | 652.86000 | NA | NA |

**Inferred ancestry of individuals:**

| **S. no.** | **Label** | **% missing** | **Inferred clusters** | | |
| --- | --- | --- | --- | --- | --- |
|  |  |  | **Pop 1** | **Pop 2** | **Pop 3** |
| 1 | Br (Rapa 12) | 0 | 0 | 0.999 | 0 |
| 2 | Br (IC 0623820) | 0 | 0 | 0.996 | 0.003 |
| 3 | Br (Pusa gold) | 0 | 0.003 | 0.911 | 0.086 |
| 4 | Bn (Nigra tall) | 0 | 0.906 | 0.004 | 0.09 |
| 5 | Bn (IC 341132) | 0 | 1 | 0 | 0 |
| 6 | Bn (IC 393266) | 0 | 0.998 | 0 | 0.002 |
| 7 | Bn (IC 399882) | 0 | 1 | 0 | 0 |
| 8 | Bn (IC 328460) | 0 | 0.999 | 0 | 0 |
| 9 | Bn (EC426390) | 0 | 0.881 | 0 | 0.118 |
| 10 | Bn (IC 338498) | 0 | 1 | 0 | 0 |
| 11 | Bn (IC 338724) | 0 | 1 | 0 | 0 |
| 12 | RBJ102 | 0 | 0.001 | 0.001 | 0.999 |
| 13 | RBJ104 | 0 | 0 | 0 | 1 |
| 14 | RBJ106 | 0 | 0 | 0 | 0.999 |
| 15 | RBJ119 | 0 | 0.001 | 0 | 0.999 |
| 16 | RBJ120 | 0 | 0.001 | 0 | 0.999 |
| 17 | RBJ122 | 0 | 0 | 0 | 1 |
| 18 | RBJ126 | 0 | 0 | 0 | 0.999 |
| 19 | RBJ128 | 0 | 0.007 | 0.005 | 0.987 |
| 20 | RBJ129 | 0 | 0.001 | 0.012 | 0.987 |
| 21 | RBJ131 | 0 | 0 | 0 | 0.999 |
| 22 | RBJ132 | 0 | 0 | 0 | 1 |
| 23 | RBJ135 | 0 | 0 | 0 | 0.999 |
| 24 | RBJ137 | 0 | 0 | 0 | 0.999 |
| 25 | RBJ141 | 0 | 0 | 0 | 1 |
| 26 | RBJ142 | 0 | 0 | 0 | 1 |
| 27 | RBJ143 | 0 | 0 | 0 | 0.999 |
| 28 | RBJ147 | 0 | 0 | 0 | 0.999 |
| 29 | RBJ148 | 0 | 0.001 | 0.274 | 0.725 |
| 30 | RBJ149 | 0 | 0 | 0.228 | 0.771 |
| 31 | RBJ150 | 0 | 0 | 0.207 | 0.792 |
| 32 | RBJ151 | 0 | 0 | 0.232 | 0.767 |
| 33 | RBJ152 | 0 | 0 | 0.215 | 0.785 |
| 34 | RBJ156 | 0 | 0 | 0.001 | 0.999 |
| 35 | RBJ159 | 0 | 0 | 0 | 0.999 |
| 36 | RBJ163 | 0 | 0 | 0.001 | 0.999 |
| 37 | RBJ166 | 0 | 0 | 0.026 | 0.974 |
| 38 | RBJ167 | 0 | 0 | 0.194 | 0.805 |
| 39 | RBJ170 | 0 | 0 | 0.566 | 0.433 |
| 40 | RBJ174 | 0 | 0.001 | 0.575 | 0.425 |
| 41 | RBJ175 | 0 | 0 | 0.99 | 0.01 |
| 42 | RBJ179 | 0 | 0 | 0.999 | 0.001 |
| 43 | RBJ186 | 0 | 0 | 1 | 0 |
| 44 | RBJ188 | 0 | 0 | 0.999 | 0 |
| 45 | P.Vijay | 0 | 0.999 | 0.001 | 0 |
| 46 | PM28 | 0 | 1 | 0 | 0 |
| 47 | Pusa Jaikisan | 0 | 0.999 | 0 | 0 |
| 48 | Varuna | 0 | 0.998 | 0 | 0.001 |
